# Inter-Observer Reliability in Myocardial Blood Flow Measurement Using Rb-82-PET/CT

**DOI:** 10.3390/tomography12080108

**Published:** 2026-07-27

**Authors:** Sebastian J. Stolte, Ole Christopher Maas, Sebastian Kopp, Hanna Geiger, Flavio Forrer

**Affiliations:** 1 Department of Radiology and Nuclear Medicine, Cantonal Hospital St. Gallen, HOCH Health Ostschweiz, 9007 St. Gallen, Switzerland; 2 Faculty of Medicine, University of Basel, 4001 Basel, Switzerland; 3 Department of Cardiology, Cantonal Hospital St. Gallen, HOCH Health Ostschweiz, 9007 St. Gallen, Switzerland

**Keywords:** myocardial blood flow, flow reserve, cardiac perfusion imaging, inter-observer reliability, Rb-82-PET/CT

## Abstract

**Background/Objectives**: Myocardial perfusion PET/CT, e.g., using Rb-82, enables the measurement of absolute myocardial blood flow (MBF) and, hence, the blood flow reserve, which plays a crucial role in the diagnosis of coronary artery disease. However, the values might depend on the observer’s analysis and, thus, depend on the observer’s experience. We investigated the inter-observer reliability of MBF measurements in Rb-82-PET/CT. **Methods**: Thirty consecutive patients who underwent Rb-82-PET/CT for suspected myocardial ischemia were analyzed. Stress conditions were simulated using 400 µg Regadenoson. MBF was measured using Corridor 4DM software. Myocardial flow reserve (MFR) is the ratio of absolute flow measurements under stress and rest conditions. Four different physicians with different levels of experience (two senior physicians and two residents) measured the MBF. The experience of the two residents was one year of experience and no previous experience, respectively. Intraclass correlation coefficients (ICC) were calculated for MBF and MFR in the three main vascular territories as well as in the whole left ventricle. Additionally, the left ventricular ejection fraction (EF) was determined. **Results**: The evaluation of MBF showed an excellent correlation between all observers, indicated by a low inter-observer variability. Comparing the two different levels of experience, senior physicians showed a slightly higher accordance in MBF assessment (ICC ranging from 0.9 to 0.98) than the residents (ICC ranging from 0.86 to 0.94). The MFR calculations showed a slightly lower reliability due to the accumulation of variability in rest and stress measurements (ICC between 0.78 and 0.81), still indicating a good reliability. The EF calculations also showed a good reliability among the different observers (ICC 0.83). **Conclusions**: Measurement of MBF, MFR and EF in Rb-82-PET/CT is highly robust with a very low inter-observer variability. A slightly higher inter-observer reliability was found for more experienced physicians.

## Simple Summary

Myocardial perfusion imaging is a major pillar of cardiological diagnostics in the field of nuclear medicine. PET-based assessments using Rb-82 as a tracer outperform conventional scintigraphic methods by offering absolute flow measurements; with this additional information, perfusion assessment becomes accessible for inexperienced users. This study primarily shows high inter-observer reliability of the method, enhancing its role as a robust tool among all levels of experience. Second, it demonstrates the reliability of measuring left-ventricular volumes and ejection fraction in comparison to echographic assessments. The study aims to enhance confidence in the method of myocardial blood flow measurement and left-ventricular volume assessment using Rb-82-PET/CT.

## 1. Introduction

With coronary artery disease (CAD) as one of the most frequent causes of mortality and invalidity, and with regard to its high impact on health costs and economics, reliable diagnostics are essential.

Initial cardiological assessment offers a decent detection rate; however, given the high prevalence, there are still many clinically unclear cases that require further investigation. According to the current European Society of Cardiology Guidelines for the management of chronic coronary syndromes, non-invasive functional imaging gains importance for patients with a moderate to high pre-test likelihood (>15–85%) for coronary artery disease [1].

From the nuclear medicine perspective, myocardial perfusion imaging using MIBI-SPECT has been a frequently used tool to detect such clinically unclear cases for many years. It offers a high sensitivity and an even higher specificity for detecting relevant myocardial ischemia. Nevertheless, the more recently developed PET/CT diagnostics offer an even higher diagnostic value, since they allow not only for visual assessment [2] but also for absolute quantification of myocardial blood flow [3,4,5] and, hence, the relative quantification of coronary reserve capacity (MFR). The examination has low side effects and is generally associated with high patient comfort [6]. For CAD of the main coronary arteries, coronary angiography remains the gold standard. However, MFR, in particular, is a valuable risk predictor and a cornerstone in the diagnostic algorithm for the diagnosis of angina or ischemia with non-obstructive coronary arteries (ANOCA or INOCA), thus guiding further therapeutic decisions [1,7,8]. PET/CT may give insight into microvascular function as well, if the myocardial blood flow is globally reduced [9]. Despite its higher costs, Rb-82-PET/CT shows a higher cost-effectiveness compared to SPECT/CT-MPI [10].

The most common radiopharmaceutical used is Rb-82, a positron emitter with a short half-life of 76 s, which is applied in the chemical form of rubidium–chloride (RbCl). Physiologically, rubidium acts as a surrogate of potassium. It is easily available because of its supply via an Sr-82/Rb-82-generator. Rb-82 is eluted from the generator as the decay product of the accelerator-produced and stannic oxide-bound Sr-82, which itself has a half-life of 25 days and 13 h. This is a major advantage compared to other perfusion tracers, such as N-13 [11] or O-15, which need an on-site cyclotron. The diagnostic power of Rb-82 is identical to O-15 and N-13, despite the slightly better kinetics and lower radiation exposure of the latter [12]. Rb-82 emits positrons with an energy of 4.401 MeV and it decays exclusively to Kr-82.

The imaging procedure is time-effective, with a total examination time of approximately 30 min and is, therefore, convenient for the patient. Because of the short half-life of Rb-82, physical exercise for stress-imaging is not feasible. Hence, physical activity is simulated with pharmacological stress, e.g., Regadenoson 400 µg intravenously [13,14,15]. For patients suffering from claustrophobia, the procedure is, therefore, more suitable than myocardial MRI; furthermore, it requires less cooperation than an MRI assessment. Rb-82 PET/CT is also feasible in patients with a pacemaker [16].

Like echocardiography or MRI, Rb-82-PET/CT can dynamically visualize left ventricular contraction and measure the ejection fraction as a surrogate for the left ventricular function. In particular, the detection of transient ischemic dilatation offers additional information for the interpretation of low MFR [17,18,19].

Calculating the myocardial blood flow and the flow reserve is a largely automated process; nonetheless, it requires individual adaptation by the conducting physician. Faulty adaptation may lead to significantly altered flow calculations and, consequently, a wrong interpretation of the exam [20,21]. Each individual intervention carries the risk of various observer-dependent errors, especially when conducted by unexperienced operators. A high inter-observer reliability has already been published; as a consequence, MFR assessment has become a commonly used tool in Rb-82-PET/CT in recent years.

The aim of this study is to confirm the high inter-observer reliability in reserve assessment in everyday practice; to assess differences between more experienced professionals and inexperienced observers; and to evaluate the inter-observer reliability in the Rb-82-PET/CT-based assessment of the ejection fraction as well as the comparability of EF measurements with echocardiography. To date, no studies have assessed whether an in-depth software training is necessary in the context of Rb-82-PET/CT perfusion imaging to obtain comparable results among operators. This study aims to answer this question and also provide confidence in the method for inexperienced users in the evaluation of daily routine data by demonstrating a high reliability when compared to experienced users [22,23].

## 2. Materials and Methods

### 2.1. Procedure and Observer Characteristics

The data of 30 consecutive patients undergoing Rb-82-PET/CT for suspected myocardial ischemia October 2022 were evaluated by four physicians. Two of these were experienced physicians with ≥15 years of experience in nuclear medicine and around 5 years of experience with Rb-82-PET/CT; two observers were residents with one year of experience in nuclear medicine and no experience at all, respectively; the latter had only performed one supervised practice session in advance. The number of patients was chosen in accordance with previous studies assessing similar questions [22,23]. Designed as a pilot study, the study population was chosen with regard to practicability and comparability to such previous analyses at the cost of slightly reduced precision and generalizability.

### 2.2. Patient Characteristics

The study included 30 patients with suspected coronary artery disease who were examined consecutively on the same scanner using the same Sr-82/Rb-82-generator. No patients were excluded; 40% of them had had prior coronary interventions (see Table 1).

### 2.3. PET/CT Protocol

Images were acquired on a 64-slice Discovery 690 scanner (GE Healthcare, Chicago, IL, USA). After acquiring a thoracic low-dose CT, 82-Rb-RbCl was applied for rest imaging using a Ruby-Fill infusion system (Jubilant DraxImage Inc., Kirkland, QC, Canada) [24]. Patients were injected with 6 MBq of Rb-82 per kg body weight [25]. Imaging took 7 min for each condition.

Stress condition was simulated using 400 µg Regadenoson applied intravenously 30 s before the second dose of 82-Rb-RbCl. The patient remained unmoved and was asked to breathe as usual during the entire examination.

### 2.4. Image Analysis

MBF was measured using Corridor 4DM v2018 software (Invia Medical Imaging Solutions, Ann Arbor, MI, USA), a commercially available software routinely used in our department and validated in various publications [26]. Each observer independently centered the myocardial signal, adjusted the alignment of the heart axes, and adapted the cardiac contours and limits both for gated and dynamic datasets. EF was automatically calculated as the ratio between minimal and maximal intraventricular volume in a stress condition.

A small ROI was positioned automatically in the left ventricle and manually adapted, if needed, to measure the arterial input. The concentration of myocardial activity in the individually set ROI was then automatically normalized to the arterial input function for the calculation of MBF. MFR was automatically calculated as the ratio between MBF in a stress condition and MBF in a rest condition, both for the left ventricular myocardium as a whole and for the single coronary regions, which were defined automatically.

### 2.5. Statistical Analysis

Intraclass correlation coefficients were calculated for MBF and MFR both in the whole left ventricle and in the single coronary regions, as well as for the ejection fraction under stress condition. We used ICC (2,1) for two-way random effects with single measures. Separate ICCs were calculated for the entire group of observers and for the different levels of experience separately. Furthermore, a Bland–Altman analysis was performed to compare PET-bases rest EF assessment and echocardiographic measurements for both the specialists and residents; the mean bias for both groups was calculated additionally. Statistical analyses were conducted using RStudio (version 2024.12).

## 3. Results

### 3.1. Total Agreement in MBF and MFR

**Figure 1:**
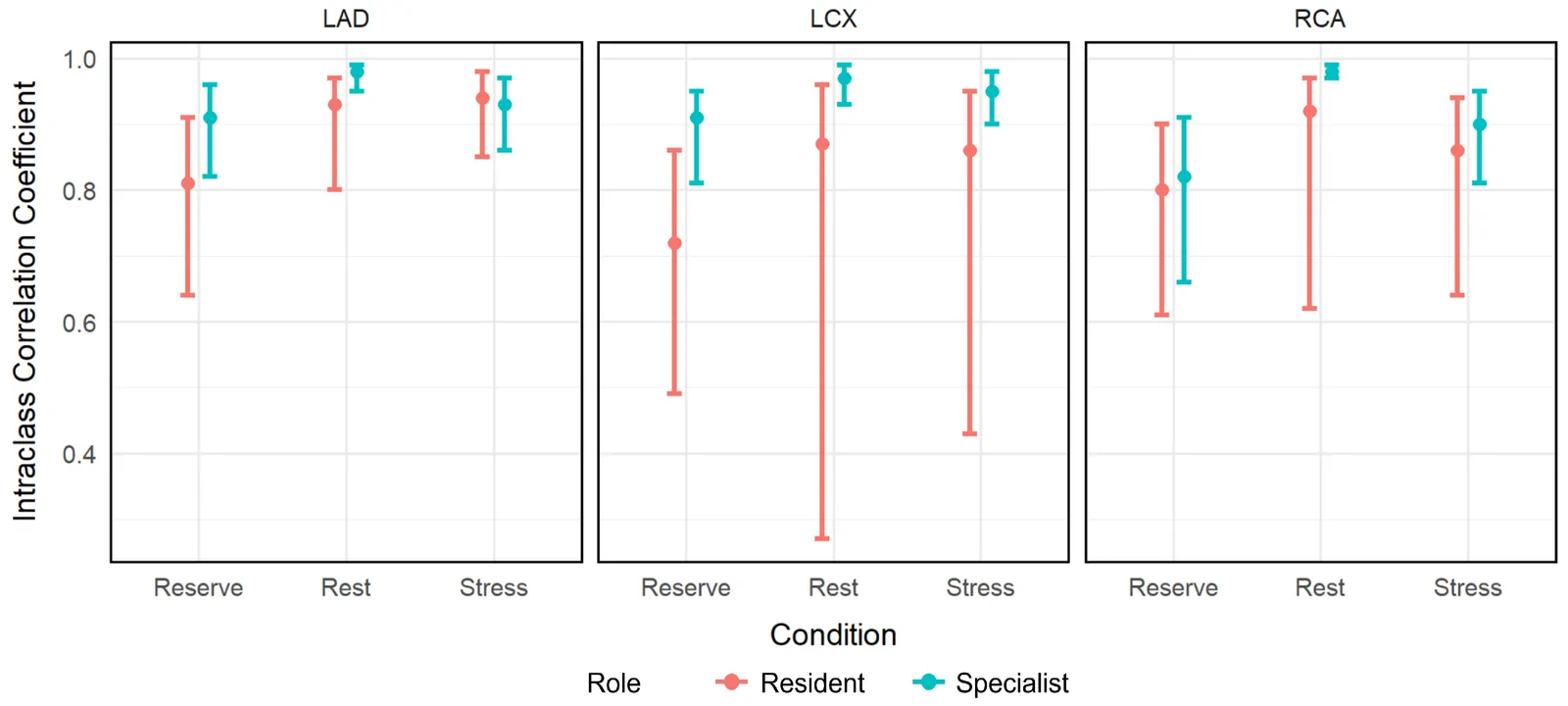
ICCs of MBF and MFR in the different territories comparing specialist ICCs versus resident ICCs.

**Figure 2:**
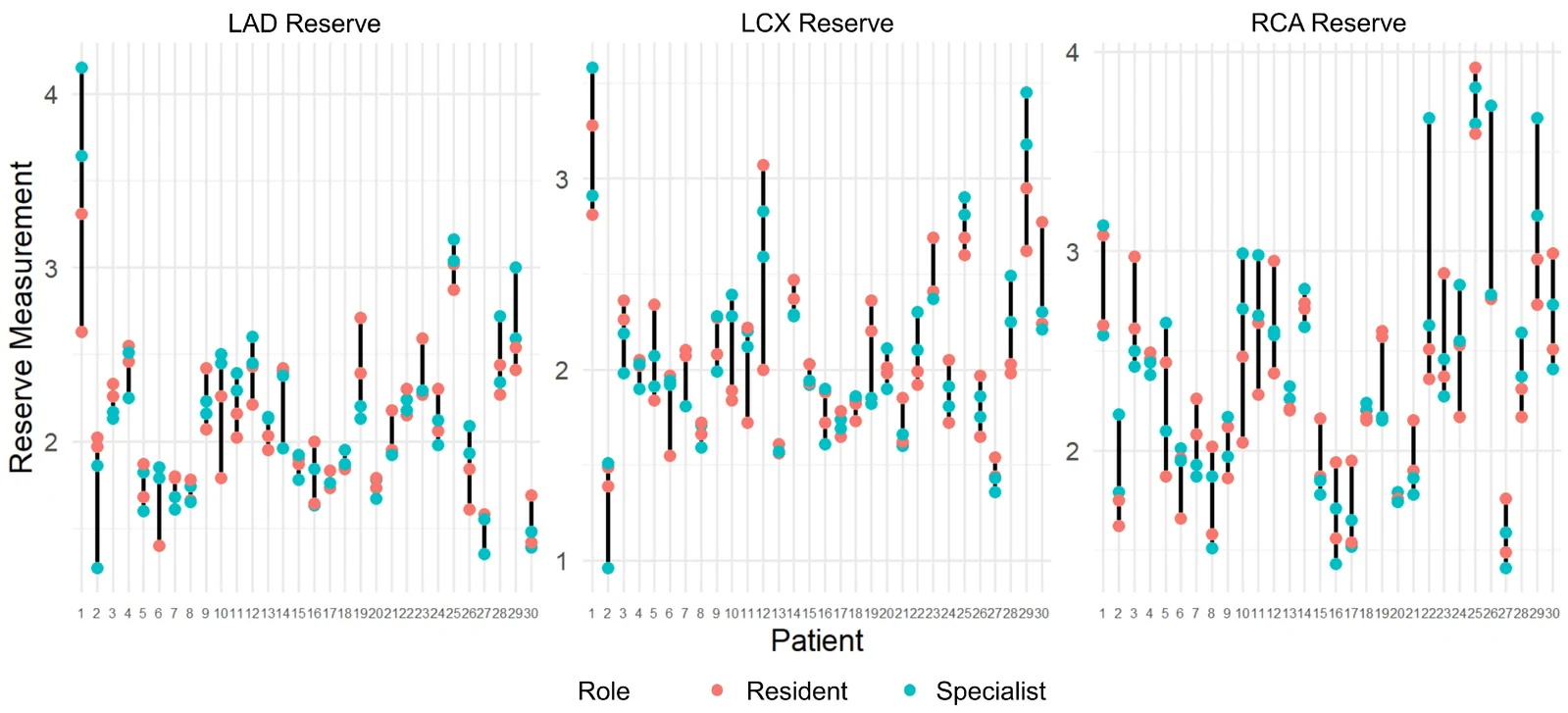
MFR Measurements of single patients for single users (two specialists and two residents).

ICC evaluation showed a generally high inter-observer agreement, with ICC levels ranging from 0.87 to 0.93 for MBF in the single vascular territories both under rest and stress conditions. The highest agreement was tendentially found in the LAD territory (see Table 2). In general, the agreement was slightly higher in the rest condition than in the stress condition. The lowest ICC was reached in the RCA territory under the stress condition. In any case, there was no significant difference in agreement for either the rest vs. stress condition or for the single vascular territories. The evaluation of the MFR agreement showed significantly lower ICC values for MFR compared to MBF. Despite this constraint, ICC values remained high for the calculated MFR as well, ranging from 0.78 to 0.81 (see Figure 1). Overall, higher disagreements were observed for higher reserve values (see Figure 2).

### 3.2. Experience-Correlated Observer Agreement for MBF and MFR

When compared to the inter-observer agreement between the residents (0.86 to 0.94), a significantly higher agreement in MBF can be observed between the specialists (0.90 to 0.98), independent of vascular territory and condition. Here again, the MFR agreement was lower than the MBF agreement for both the specialists and the residents. Despite being lower than the specialist agreement, the agreement between the residents was still high.

### 3.3. Observer Agreement in EF and Correlation to Echocardiographic Pre-Assessments

For the EF evaluation, high ICC values demonstrated a high inter-observer agreement. Contrary to the evaluation of MBF and MFR, residents showed a higher inter-observer agreement, in this case, than the specialists, with only a moderate agreement among the latter (ICC 0.74 versus 0.91 between residents). The variation seems quite high among both the specialists and residents, with considerably overlapping confidence intervals.

**Figure 3:**
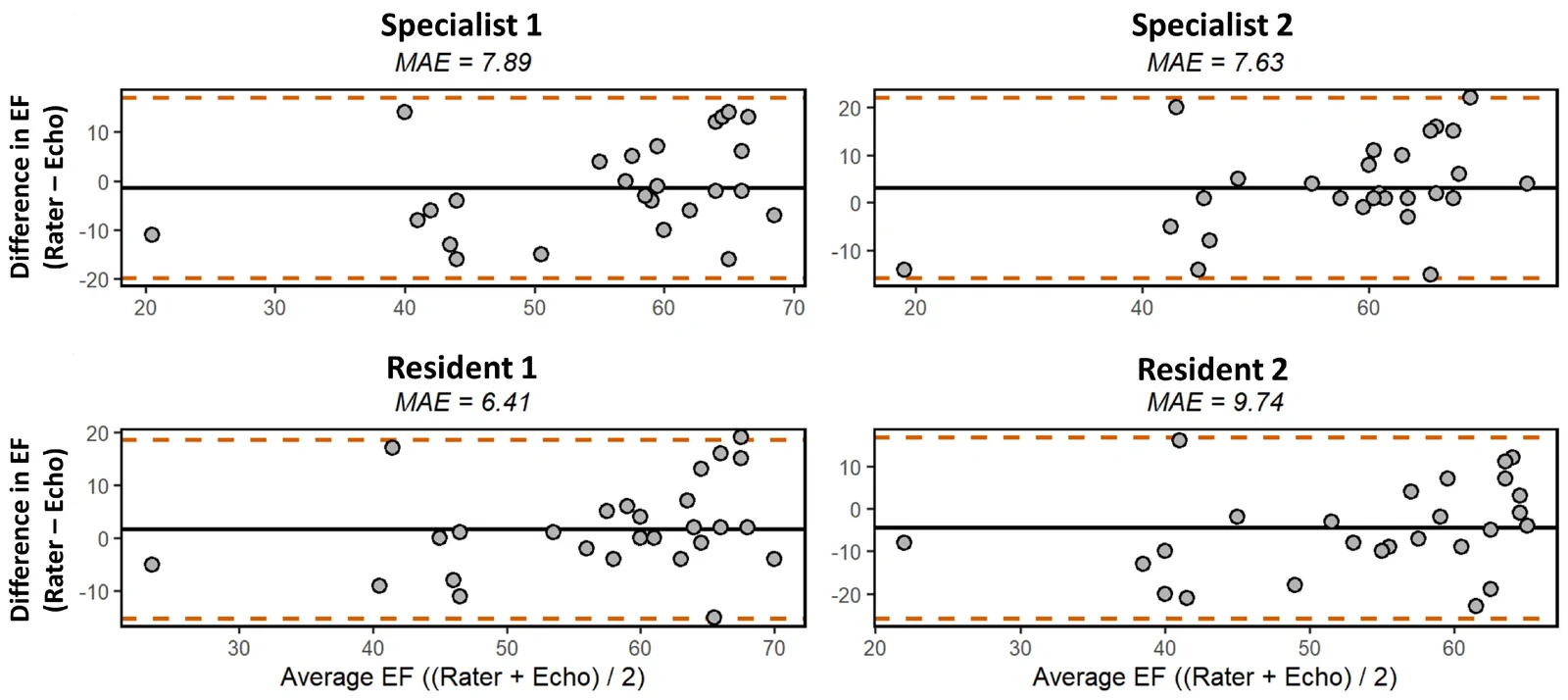
Bland–Altman analysis for EF values. MAE: mean absolute error. Circles: Single deviations of individual measurements from the echocardiographic measurement. Solid line: mean deviation from echocardiographic measurement. Dashed lines: 95% confidence interval.

In 27 out of 30 patients, echocardiographic rest EF measurements were available. The Bland–Altman analysis for the comparison of EF measurements with Rb-82-PET and echocardiography shows comparable mean absolute errors for all four operators (between 6.41 and 9.74%, see Figure 3); these differences among single operators are comparable to the differences in EF evaluations based on echocardiography [27]. Overall, the mean bias for both groups (specialists versus residents), when compared to echocardiography-based EF assessment, was 0.9% (95% CI: −1.7–3.6%) and −1.4% (95% CI: −4.1–1.4%), respectively.

## 4. Discussion

To our knowledge, this is the first study investigating the inter-observer reliability between physicians at different levels of experience in the setting of Rb-82-PET-based perfusion imaging for daily routine patient data. Furthermore, we can confirm the high inter-observer reliability for the measurement of MBF and MFR using Rb-82-PET in our setting, verifying other studies on the method’s reliability. These findings support previously published results that recommend MBF and MFR as additional important parameters in the assessment of myocardial ischemia. We prove the feasibility of these measurements in daily clinical routines. Only slight differences between residents with low experience and specialists were found, which again proves the robustness of the method. Indeed, the highest accordance was found among experienced specialists, which might indicate that a certain level of experience could be favorable, despite the overall very high accordance. Thus, despite a small effect of training on the reliability, there is no obstacle to involving unexperienced users in MFR assessment early on in their residencies.

We found a higher inter-observer reliability of MBF assessment compared to MFR assessment. Moderately higher discrepancies could be seen in the MFR assessment compared to MBF. MFR assessment seems to be more prone to inter-observer variation, since it accumulates the variation of rest and stress MBF assessment as a calculated value. The conclusion of Klein et al. suggests that the difference between MBF in stress and rest conditions (hence the absolute increase of perfusion instead of a relative increase) provides a slightly more stable value in the context of inter-observer reliability. Nevertheless, this higher variation in MFR assessment does not have a clinical impact, since the highest discrepancies among observers could be seen with rather high MFR values, clearly above the clinically relevant cut-off of MFR 1.8, below which patients benefit significantly from revascularization [28]. In our study, lower ICC values in MFR assessment were mostly found in an MFR range > 2.5.

Clinically, these findings are important, since they prove the high inter-observer reliability in daily routine diagnostics and show that no extensive training or experience is needed for the calculation of Rb-82-based perfusion measurements. It provides confidence in the method for unexperienced users and shows that the method can be applied without strict supervision by more experienced physicians.

EF assessment also showed a good inter-observer reliability. Nonetheless, the accordance among observers was significantly lower compared to MBF and MFR assessments, most likely because the definition of the left ventricular basis allows for more variability but is crucial for volume calculation. Retrospectively, residents seemed to be more confident in the automatic determination of the myocardial basis while more experienced users tended to define the basis by themselves [29,30]. Regarding the broad confidence intervals of EF ICCs among seniors and residents, the relevance of this higher user-dependent variability among experienced users compared to a more automated processing among residents might be of less relevance.

PET/CT-based EF calculation is only another add-on in examinations conducted to detect advanced hypokinetics and seriously impaired left-ventricular function. PET/CT lacks the accuracy of MRI in measuring left-ventricular volume and ejection fraction, since the valvular level is not reliably represented in the images with the perfused muscle and acts only as a surrogate for the dimension of left-ventricular volume. As mentioned, most patients receive echocardiography prior to an Rb-82-PET/CT; consequently, hypokinetics and impaired ventricle function are already known in most cases. In our study population, 90% of patients had prior echocardiographic examinations. Our comparison between rest EF measurements in Rb-PET/CT and in echocardiography showed similar deviations among specialists and residents, as was expected with respect to the good ICC values. In general, the deviation in echocardiographic measurements was, nevertheless, fairly high, with low agreement between the two methods. PET/CT measurements seemed to offer only an estimation of the overall left ventricular function. However, there was no hint of a systematic error, with a very low mean bias for both groups and without significant differences among specialists and unexperienced users. It needs to be mentioned that echocardiography is also subject to inter-observer variability and cannot be regarded as the gold standard. That is why the inter-observer reliability of PET/CT-based EF assessment seems comparable to that of the echography-based EF assessment in our study.

In this study, a dataset of 30 consecutive patients was acquired to obtain a patient collective as random and as clinically representative as possible. The relatively small population size might negatively affect the precision and generalizability of the results, especially when regarding the broad confidence intervals; this limitation seems inferior regarding the pilot study character and the otherwise explicit results of the study. Rb-82-application was conducted using a newly installed generator to minimize differences in the activity concentration of the injected radiopharmaceutical.

Intra-observer reproducibility was not examined in the present study; other studies have already addressed this issue, demonstrating the high level of inter-observer reliability of the method in both experienced and in inexperienced operators [22].

Being conducted as a single-center study evaluating only one PET/CT scanner and one single software package, the results cannot be universalized; nevertheless, we assume that comparable scanners with different routinely used and authorized software packages would deliver similar findings; similar studies might be conducted under different circumstances to prove this assumption.

In our institute, the method is not only used in patients with a high clinical suspicion of myocardial ischemia but also as an option to exclude ischemia in patients where ergometry is not possible. Therefore, only a small percentage of patients with relevantly impaired MFR and hints for ischemia in Rb-82-PET/CT were included. Secure statements on the actual inter-observer reliability, specifically in the crucial MFR range of around 1.8 [31] are, therefore, not possible with our data. According to our findings, the method nevertheless seems to show a very high inter-observer reliability in this range; after all, the highest discrepancies were found in higher MBF and MFR ranges. Consequently, a comparison with coronary arteriography as a gold standard for coronary artery assessments of the main coronary arteries is also not realistically possible regarding the low percentage of patients with relevant ischemia who afterwards received coronary angiography. Meanwhile, from the perspective that INOCA and ANOCA are coming more and more into the clinical focus, it is mandatory to have a robust non-invasive, operator-independent method with which to measure MBF. It might be interesting to plan a further study with pre-selected patients that have a proven pathological coronary artery calcium score [32,33].

## 5. Conclusions

The measurement of MBF and MFR in Rb-82-PET/CT has an excellent inter-observer reliability, which confirms previously published data [22,23,34] and demonstrates the robustness of the method. To our knowledge, this is the first study that showed a slightly higher inter-observer reliability for more experienced physicians. The only discrete training benefit for the inter-observer accordance demonstrates the minor effect of experience for the determination of MBF and MFR, which does not seem clinically relevant regarding the overlapping confidence intervals. The accordance among users was higher for the MBF assessment than for the MFR, since the latter is a calculated value made on the basis of rest and stress MBF accumulating inter-observer variabilities of these two measurements. Nevertheless, this effect does not seem to have a clinical relevance regarding the specifically high inter-observer reliability for clinically relevant MFR values < 2.5.

MBF and MFR measurement in Rb-82-PET/CT remain highly reliable tools in the assessment of coronary ischemia.

EF measurements show slightly higher inter-observer variations, suggesting that this variable provides only secondary information from Rb-82-PET/CT, with MRI and echocardiography being more reliable tools. Nevertheless, EF measurements also showed a high inter-observer reliability, indicating the robustness of the method; however, there were considerable deviations from EF measurements in echocardiography. In general, EF measurement in Rb-82-PET/CT offers a rough estimation of left ventricular ejection fraction and remains a useful tool for major left-ventricular function impairment.

## Figures and Tables

**Table 1:** Patient characteristics. Mean +/− standard deviation; the values for EF, MBF and MFR are based on the clinical report for which the observers were blinded in this study.

Sex—male	15/30
Age	68.2 +/− 10 years
Height	166 +/− 9.4 cm
Weight	82.5 +/− 18.3 kg
BMI	30.1 +/− 6.3 kg/m^2^
Prior coronary interventions	12/30
Echocardiographic EF available	27/30
Agatston-Score in native vessel-patients	234 +/− 598 pt.
SSS	2.7 +/− 5.4
SDS	0.8 +/− 1.4
Rest EF	55.3 +/− 12.9%
Stress EF	60.8 +/− 13.4%
Rest MBF	0.8 +/− 0.3 mL/min/g
Stress MBF	1.7 +/− 0.7 mL/min/g
MFR	2.2 +/− 0.5

BMI: body mass index; SSS: summed stress score; SDS: summed differential score; EF: left ventricular ejection fraction; MBF: myocardial blood flow; MFR myocardial flow reserve. Informed consent was obtained from all patients.

**Table 2:** ICC calculations for MBF and MFR in the different vascular territories as well as for EF determination for all observers together versus ICC among specialists and residents separately.

	Condition	Total ICC	Specialist ICC	Resident ICC
	Rest	0.93 [0.87, 0.97]	0.98 [0.95, 0.99]	0.93 [0.80, 0.97]
LAD	Stress	0.93 [0.87, 0.96]	0.93 [0.86, 0.97]	0.94 [0.85, 0.98]
	Reserve	0.81 [0.70, 0.89]	0.91 [0.82, 0.96]	0.81 [0.64, 0.91]
	Rest	0.91 [0.78, 0.96]	0.97 [0.93, 0.99]	0.87 [0.27, 0.96]
LCX	Stress	0.91 [0.80, 0.96]	0.95 [0.90, 0.98]	0.86 [0.43, 0.95]
	Reserve	0.81 [0.70, 0.89]	0.91 [0.81, 0.95]	0.72 [0.49, 0.86]
	Rest	0.93 [0.87, 0.97]	0.98 [0.97, 0.99]	0.92 [0.62, 0.97]
RCA	Stress	0.87 [0.79, 0.93]	0.90 [0.81, 0.95]	0.86 [0.64, 0.94]
	Reserve	0.78 [0.66, 0.88]	0.82 [0.66, 0.91]	0.80 [0.61, 0.90]
EF	Stress	0.83 [0.73, 0.91]	0.74 [0.53, 0.87]	0.91 [0.57, 0.97]

## Data Availability

Data are available on request from the authors. More information can be obtained at https://zenodo.org/records/21495375, accessed on 22 July 2026.

## Abbreviations

The following abbreviations are used in this manuscript:

MBF	myocardial blood flow
MFR	myocardial flow reserve
ICC	intraclass- correlation coefficient
EF	left ventricular ejection fraction
CAD	coronary artery disease
ANOCA	angina with non-obstructive coronary arteries
INOCA	ischemia with non-obstructive coronary arteries
MPI	myocardial perfusion imaging
RbCl	rubidium-chloride
BMI	body mass index
SSS	summed stress score
SDS	summed differential score
ROI	region of interest
LAD	left anterior descending artery
RCA	right coronary artery
LCX	left circumflex artery
MAE	mean absolute error
